# Process Evaluation of Workplace Interventions with Physical Exercise to Reduce Musculoskeletal Disorders

**DOI:** 10.1155/2014/761363

**Published:** 2014-12-10

**Authors:** Lars L. Andersen, Mette K. Zebis

**Affiliations:** ^1^National Research Centre for the Working Environment, Lersø Parkalle 105, 2100 Copenhagen Ø, Denmark; ^2^Faculty of Health and Technology, Metropolitan University College, Sigurdsgade 26, 2200 Copenhagen N, Denmark

## Abstract

Process evaluation is important to explain success or failure of workplace interventions. This study performs a summative process evaluation of workplace interventions with physical exercise. As part of a randomized controlled trial 132 office workers with neck and shoulder pain were to participate in 10 weeks of elastic resistance training five times a week at the workplace; the 2 min group performed a single set of lateral raise to failure, and the 12 min group performed 5-6 sets with 8–12 repetitions. Participants received a single instructional session together with a training diary and manual at baseline (100% dose delivered and 100% dose received), and 59 and 57 participants, respectively, replied to the process evaluation questionnaire at 10-week follow-up. Results showed that in the 2 and 12 min groups, respectively, 82% and 81% of the participants completed more than 30 training sessions. However, two-thirds of the participants would have preferred more than a single exercise to vary between. In the 12 versus 2 min group more participants experienced the training sessions as too long (30% versus 5%). Most participants (67–92%) found the training diary and manual helpful, adequacy in a single instructional session, and satisfaction with the type of training. Among those with low adherence, lack of time (51%) and difficulties in starting exercising after illness (26%) were common barriers for regular training. Among those with low adherence, 52% felt that five training sessions per week were too much, and 29% would rather have trained a completely different kind of exercise. In conclusion, resistance training at the workplace is generally well received among office workers with neck-shoulder pain, but a one-size-fits-all approach is not feasible for all employees.

## 1. Introduction

Musculoskeletal disorders are common and costly in the working population across Europe as well as in the United States [1, 2]. A Danish survey among the general working population found that almost a third of white-collar workers suffered from pain in the neck and shoulders, which was associated with 35% increased risk of long-term sickness absence [3]. In addition to the cost for workplaces and society, musculoskeletal pain often has long-term adverse physical and psychological consequences for the individual [4]. Among office workers, the majority of neck and shoulder pains manifest as moderate to severe muscle tenderness in the trapezius, neck extensors, levator scapulae, and infraspinatus muscles [5]. Interventions to reduce neck and shoulder pain have therefore focused on either relaxing the painful muscles [6] or performing physical exercise to strengthen them [7].

Research on physical exercise as active intervention to reduce neck and shoulder pain has gained increasing focus during recent decades. Some of the first high-quality randomized controlled trials showed contrasting results on the effectiveness of dynamic muscle training or specific strength training on neck pain [8, 9], which can be difficult to explain without a rigorous process evaluation. In general there are promising effects of strength and endurance training on pain in the neck and shoulders [7, 10–15], but effect sizes as well as adherence to the interventions have varied. In this regard, the workplace may be an optimal social setting to encourage and perform physical exercise and other health promoting activities together with colleagues [16]. However, while most studies with physical exercise at the workplace have reported on effectiveness of the interventions, few have performed process evaluation [17].

Process evaluations are important to explain the mechanisms of success or failure of workplace interventions [17–20]. Components of process evaluations of workplace interventions have often included recruitment, reach, fidelity, satisfaction, dose delivered, dose received, barriers, and facilitators [17, 21–23]. Evaluation of interventions can be formative or summative in nature. While formative evaluations utilize ongoing feedback to continuously check and adjust progress of interventions, summative evaluations analyze data at follow-up to evaluate whether the intervention was implemented as intended and to provide guidance for future interventions [23–26]. This study performs a summative process evaluation of two brief daily resistance training programs for neck and shoulder pain among office workers.

## 2. Methods

### 2.1. Study Design and Population

Data for this study was obtained from a randomized controlled trial published elsewhere [27]. The primary outcome of the previously published trial showed that the 2 min and 12 min groups significantly and to a similar extent reduced pain and tenderness in the neck and shoulders [27]. The randomized controlled trial included 198 office workers with frequent pain in the neck and shoulders during the last year and tenderness of the neck-shoulder muscles. Using a computer-generated random numbers table, an independent statistician performed the concealed random allocation of participants stratified for gender and workplace. The statistician performed this procedure following the baseline examination of all participants and then informed the participants via email about group allocation and stored the randomization codes in a sealed opaque envelope until the study ended. Of the 198 participants of the randomized controlled trial, 132 participated in the two groups with physical exercise. At follow-up 128 of these replied to the questionnaire on pain (primary outcome published elsewhere [27]) and 116 replied to the process evaluation questionnaire used in the present analysis.  Table 1 shows the baseline characteristics of these 116 participants.

Participants were informed about the main objective and content of the project and gave written informed consent to participate in the study which conformed to The Declaration of Helsinki and was approved by the Local Ethical Committee (HC2008103) (trial registration: http://www.isrctn.com/ISRCTN60264809).

### 2.2. Intervention

A requirement was that the program should be possible to implement at workplaces. To eliminate the need for training machines, weights, and a gym, each participant received a set of elastic resistance tubing (TheraBand, Hygenic Corporation, Akron, Ohio). Compared with dumbbells the red, green, and blue resistance tubings correspond to approximately 2, 3, and 4 kg, respectively [28]. Participants were recommended to train at the workplace during workdays, and because most employees worked from Monday to Friday this corresponded to five times a week during the 10-week intervention. The program built on the principle of progressive overload [29].

The 2-minute group performed the exercise “lateral raise,” that is, shoulder abductions in the scapular plane, in a slowly controlled manner for a single set to momentary muscular fatigue, that is, with as many consecutive repetitions as possible without pause between repetitions. During the first two weeks, women used red elastic tubing and men used green elastic tubing. Participants were asked not to increase the resistance level for the first two weeks, only repetitions. During each training session, participants were to attempt to break their own previous record in terms of repetitions. However, they were to terminate the set if they could perform repetitions for more than two minutes. After two weeks participants progressed to a higher level of resistance, again receiving instructions from the manual to increase resistance when they could perform more than a specified number of repetitions according to the following scheme: 22, 20, 18, and 16 repetitions, respectively, at the 2nd (green for women, blue for men), 3rd (blue for women, green + red for men), 4th (green + red for women, blue + red for men), and 5th (blue + red for women, blue + green for men) levels of resistance.

The 12-minute group performed the same exercise as the 2 min group, that is, lateral raise, but performed 5-6 sets of 8–12 repetitions. During the first two weeks, women used red elastic tubing and men used green elastic tubing. Participants were asked not to increase the resistance level for the first two weeks, only repetitions and sets. After two weeks, they progressed to a higher level of resistance (if they could complete 6 sets of 12 repetitions) and followed instructions to increase resistance again when they could complete 6 sets of 12 repetitions with the new color of resistance. They were to begin new sets approximately every other minute, completing their training sessions in 12 minutes.

In each intervention group separately, therapists provided an initial instructional session on the overall program and on how to correctly perform the exercises during a 30-minute group meeting. Participants had five different days to choose between, and between 5 and 15 participant showed up at each session. Subsequently, participants performed the exercise unsupervised and registered all training in a training diary. While subsequent training was unsupervised, optional help (by email and telephone) with the program was available from the therapists throughout the intervention period.

### 2.3. Context of the Intervention

The intervention was performed at two large office workplaces with several departments in Copenhagen, Denmark. The participants were able to perform the elastic resistance exercises at the offices or in the hallways, which eliminated the need for transportation to a gym. The upper management approved and supported the intervention by announcing that employees could participate during paid working hours. This message was delivered through the company's intranet as well as by an email to all employees.

### 2.4. Process Evaluation Components and Definitions

According to previous workplace intervention studies the following components are recommended to be included in process evaluations: recruitment, reach, fidelity, satisfaction, dose delivered, dose received, barriers, and facilitators [17, 21–23]. However, as the definition and use of these terms have varied between studies, likely due to differences in design, study populations, and type of interventions, we define the terms used in the present study as follows.

Reach can be defined as the percentage of the intended audience that participates in the study. In the present study, the intended audience was those with frequent neck-shoulder pain and without contraindications for participation [27]. Due to the nature of the present study, we only had information on initial eligibility through the screening questionnaire. Thus, we defined reach as the percentage of the initially eligible participants who replied “yes” to participation on the screening questionnaire and subsequently showed up for the invited clinical examination.

Dose delivered can be defined as the percentage of the intended instructional sessions that was provided by the physical therapists. In the present study, only one instructional session per participant was intended, however offered at five different days.

Dose received can be defined in two ways, (1) the percentage of participants showing up for the instructional session and (2) the number or percentage of training sessions completed during the 10 weeks. In the present study, we chose to use the first as a measure of dose received and included the latter in the fidelity component.

Fidelity can be defined as to which extent the intervention was implemented as planned. In the present study, participants implemented training themselves after the initial instructional session. Thus, we chose to evaluate  fidelity based on the participants training diary registrations as explained in detail in the following. The reason for this is that the actual number of training sessions, repetitions, and sets used in each group during the 10 weeks is a good indicator to whether the participants were able to understand and implement the intended training program.

Satisfaction can be defined as the workers attitude towards the intervention [22] or level of satisfaction on a 10-point scale [21]. In the present study with a very specific and simple training program, we customized questions on satisfaction, barriers, and facilitators in the follow-up questionnaire as described in the following.

### 2.5. Training Diary Registrations (Fidelity)

**Figure 1:**
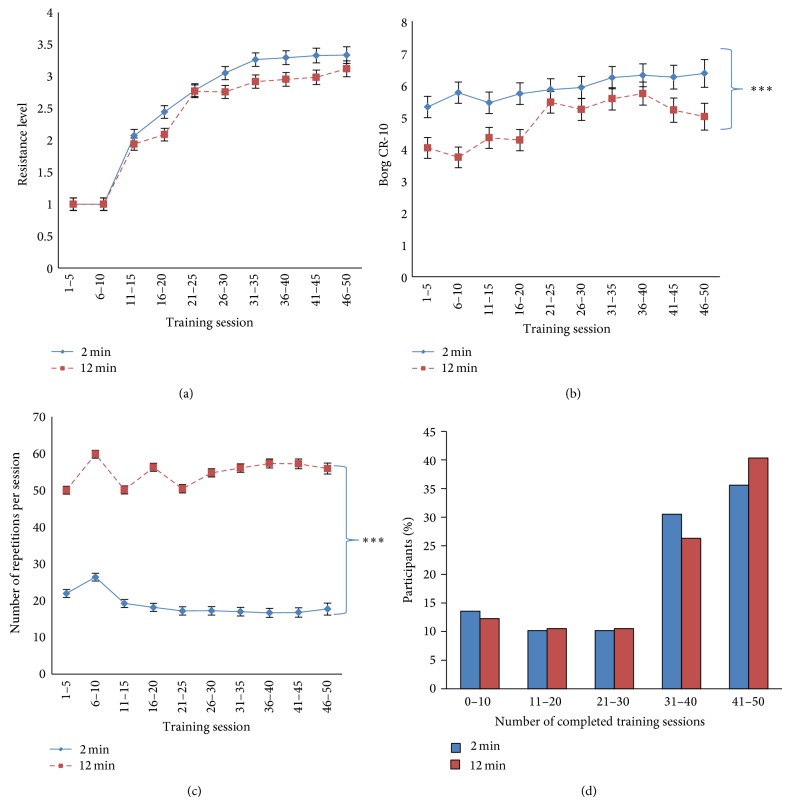
Training diary registrations (fidelity). Progression of resistance (a), physical exertion during training (b), repetitions per training session (c), and percentage of participants completing different number of training sessions (d) in the 2 and 12 min groups. Values are least square means (SE) or percentage of participants. ^***^Significant group effect (linear mixed model, *P* < 0.001).

During each training session, participants noted number of sets and repetitions, resistance level (red (easy), green (medium), and blue (hard) elastic tubing), and physical exertion (Figure 1). In the present study where participants trained without supervision after the initial sessions, this information was used to evaluate whether participants followed the intended interventions (fidelity). For women and men, respectively, the red and green elastic tubings were defined as the first resistance level. Participants could also add the colors in parallel to gain more resistance if the highest resistance level (blue) became too easy. Participants also noted perceived physical exertion during the last repetition of each training session using the Borg-CR10 scale [28].

### 2.6. Follow-Up Questionnaire (Satisfaction, Barriers, and Facilitators)

At 10-week follow-up participants replied to questions on (1) characteristics of the training program, (2) the type of training, (3) the supportive elements related to the training program, and (4) reasons for missing exercise sessions. 

**Figure 2:**
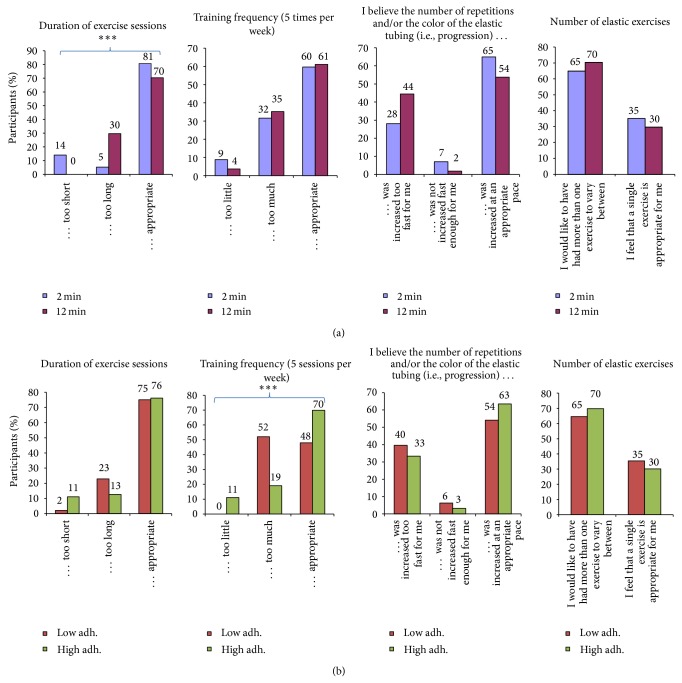
(a) Follow-up questionnaire (satisfaction, barriers, and facilitators). Participant feedback on the characteristics of the training program, that is, duration of exercise sessions (1st panel), training frequency (2nd panel), progression (3rd panel), and number of exercises (4th panel) in the 2 and 12 min groups. ^***^Significant difference (Fisher's exact test, *P* < 0.001). (b) Follow-up questionnaire (satisfaction, barriers, and facilitators). Participant feedback on the characteristics of the training program, that is, duration of exercise sessions (1st panel), training frequency (2nd panel), progression (3rd panel), and number of exercises (4th panel) among those with low and high adherence. ^***^Significant difference (Fisher's exact test, *P* < 0.001).

*Characteristics of the Training Program (Figures 2(a) and 2(b)).* To gain more understanding about the perception of the specific program, participants of each group were asked (i) whether the duration of the exercise sessions was too short, too long, or appropriate, (ii) whether the intended training frequency of five times per week was too little, too much, or appropriate, (iii) whether the progression (i.e., number of repetitions and/or resistance level) was increased too fast, not increased fast enough, or increased at an appropriate pace, and (iv) whether they would like to have had more than one exercise to vary between or if a single exercise was appropriate.

**Figure 3:**
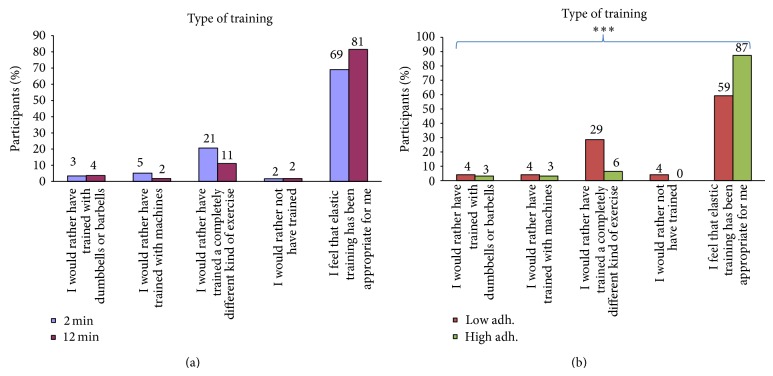
(a) Follow-up questionnaire (satisfaction, barriers, and facilitators). Participant feedback on the type of training in the 2 and 12 min groups. (b) Follow-up questionnaire (satisfaction, barriers, and facilitators). Participant feedback on the type of training among those with low and high adherence. ^***^Significant difference (Fisher's exact test, *P* < 0.001).

*Type of Training (Figures 3(a) and 3(b)).* Participants of each group were also asked whether the type of training (i.e., elastic resistance training) was appropriate for them or if they would have liked to train some other way (with the following options: dumbbells or barbells, training machines, and trained a completely different kind of exercise) or not trained at all. 

**Figure 4:**
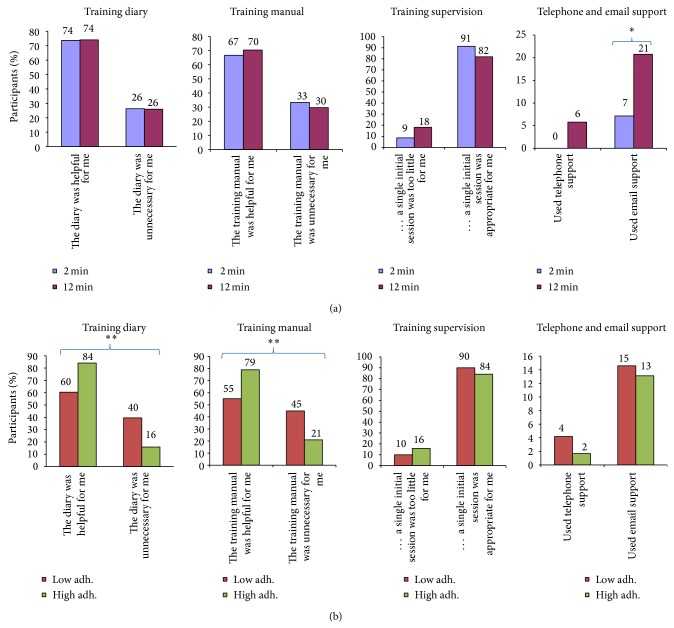
(a) Follow-up questionnaire (satisfaction, barriers, and facilitators). Participant feedback on supportive elements related to the training program, that is, the training diary (1st panel), training manual (2nd panel), training supervision (3rd panel), and telephone and email support (4th panel) in the 2 and 12 min groups. (b) Follow-up questionnaire (satisfaction, barriers, and facilitators). Participant feedback on supportive elements related to the training program, that is, the training diary (1st panel), training manual (2nd panel), training supervision (3rd panel), and telephone and email support (4th panel) among those with low and high adherence. ^**^Significant difference (Fisher's exact test, *P* < 0.01).

*Supportive Elements (Figures 4(a) and 4(b)).* Participants of each group were also asked about the supportive elements related to the training program. Specifically they were asked (i) whether the training diary was helpful or unnecessary, (ii) whether the training manual was helpful or unnecessary, (iii) whether the training supervision, that is, a single initial instructional session, was too little or appropriate, and (iv) whether they had used the optional email support and telephone support, respectively. 

**Figure 5:**
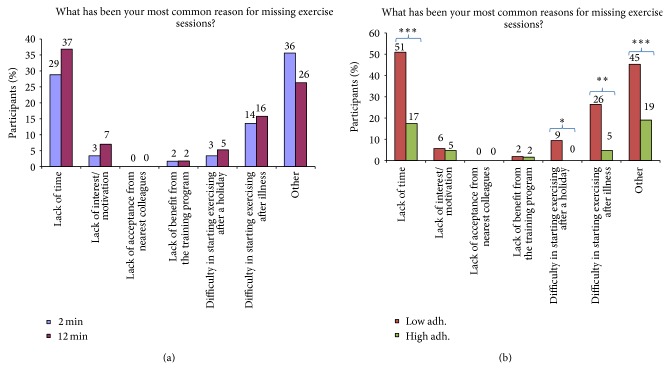
(a) Follow-up questionnaire (satisfaction, barriers, and facilitators). Reasons for missing exercise sessions in the 2 and 12 min groups (multiple-choice question). (b) Follow-up questionnaire (satisfaction, barriers, and facilitators). Reasons for missing exercise sessions among those with low and high adherence (multiple-choice question). ^*^^,^^**^^,^^***^Significant difference (Fisher's exact test, *P* < 0.05, 0.01, 0.001, resp.).

*Barriers (Figures 5(a) and 5(b)).* Participants of each group were also asked in a multiple-choice question for the most common reasons for missing exercise sessions with the following reply options: lack of time, lack of interest/motivation, lack of acceptance from nearest colleagues, lack of benefit from the training program, difficulty in starting after a holiday, difficulty in starting exercising after illness, or other reasons.

### 2.7. Statistics

For statistical analyses of the training diary registrations (resistance level, perceived exertion, and number of repetitions, resp.) a linear mixed model was used (Proc Mixed, SAS). Group (2 min, 12 min), session (0–50 training diary registrations), and group by session interaction were entered as fixed factors. Participant was entered as repeated factor. The estimation method was restricted maximum likelihood (REML) with degrees of freedom based on the Kenward-Roger approximation [30]. Only *P* values from the main effects are reported, but the least square means and standard errors are used to graphically illustrate the development over time (Figure 1).

For statistical analyses of the follow-up questionnaire, Fisher's exact test (Proc Freq, SAS) was used to test for differences in the replies between the 2 and 12 min groups as well as between those with low and high adherence to training (for the 2 and 12 min groups combined). To ensure an adequate number of participants in each adherence group and thus adequate statistical power, low and high adherence were defined as those completing less than and equal to or higher than the median number of training sessions during the 10 weeks, respectively (dichotomization). This resulted in 53 and 63 participants in the low and high adherence groups, respectively.

SAS version 9.3 was used for all analyses (SAS institute, Cary, NC). *P* values of 0.05 or less were accepted as statistically significant.

## 3. Results

Table 1 shows demographics and clinical and work-related characteristics of the participants. In general, the duration and intensity of pain in the neck and shoulders were high, and participants spent most of their working time at a computer. The 59 and 57 participants who replied to the follow-up process evaluation questionnaire were not significantly different from the 66 and 66, respectively, who were randomized at baseline (statistical comparison not shown). However, the number of completed training sessions among the 7 and 9 participants who did not reply to the process evaluation questionnaire at follow-up was only 11 (SD 13) and 15 (SD 10) in the 2 and 12 min groups, respectively.

### 3.1. Recruitment, Reach, Dose Delivered, and Dose Received

Recruitment was two-phased and consisted of a screening questionnaire and a clinical examination. The screening questionnaire was emailed to 1094 employees of whom 653 responded (60%). Of the respondents, 368 could be defined as neck-shoulder pain cases. Among the 368 neck-shoulder pain cases, 305 replied that they were willing to participate in the study and therefore they were invited for a clinical examination. Of the 305 invited, 258 showed up for the clinical examination. Thus, reach was 70% (i.e., 258 out of 368 neck-shoulder pain cases). Because we have no health related information on the 441 employees who did not respond to the screening questionnaire, the lowest theoretical reach would be 32% if all 441 nonrespondents were neck-shoulder pain cases (258 out of 368 + 441).

Dose delivered was 100%; that is, the therapists delivered all planned introductory sessions. Dose received was 100%; that is, all participants showed up for the introductory session.

### 3.2. Training Diary Registrations (Fidelity)

Figure 1 shows the progression of resistance, physical exertion, number of repetitions, and completed training sessions in the 2 and 12 min groups. After the initial two weeks there was a rapid progression in the level of resistance in both groups for about 5 weeks, whereafter the progression levelled off (main effect of session, *P* < 0.001). In both groups, the resistance was more than doubled during the 10 weeks, that is, from an average of level 1 (red tubing for women) to an average of level 3 (blue tubing for women). The level of perceived physical exertion also increased during the 10 weeks (main effect of time, *P* < 0.001), and the 2 min group experienced significantly higher physical exertion during training than the 12 min group (main effect of group, *P* < 0.001). The total number of repetitions per training session was higher in the 12 min group than in the 2 min group (main effect of group, *P* < 0.001). The number of completed training sessions was high and not significantly different between the groups, and most participants performed more than 30 training sessions during the 10 weeks (82% and 81% in the 2 and 12 min groups, resp.).

### 3.3. Follow-Up Questionnaire (Satisfaction, Barriers, and Facilitators)

Figures 2(a) and 2(b) show participants feedback on the characteristics of the training program ((a) 2 versus 12 min and (b) low versus high adherence). Significantly more participants of the 12 than 2 min group experienced the duration of the exercise sessions as too long (30% versus 5%, *P* < 0.001). For both groups together, approximately a third of the participants felt that the progression was too fast, and two-thirds would have liked to have more than one exercise to vary between them. Among those with low adherence compared with high adherence, significantly more participants felt that 5 training sessions per week were too much (52% versus 19%, *P* < 0.001).

Figures 3(a) and 3(b) show participants feedback on the type of training ((a) 2 versus 12 min and (b) low versus high adherence). For both the 2 and 12 min groups, approximately every 3 of 4 participants felt that elastic resistance training had been appropriate for them. Among those with low adherence compared with high adherence, significantly fewer participants felt that elastic resistance training had been appropriate for them (59% versus 87%, *P* < 0.001), and 29% would rather have trained a completely different kind of exercise.

Figures 4(a) and 4(b) show participants feedback on the supportive element of training ((a) 2 versus 12 min and (b) low versus high adherence). In general, most participants of the 2 and 12 min groups found that the training diary and manual were helpful and that a single instructional session was adequate. Among those with low adherence compared with high adherence significantly more participants felt that the training diary and manual were unnecessary (40–45% versus 16–21%, *P* < 0.01). Few participants had used the telephone support, but in the 12 versus 2 min group significantly more participants had used the email support (21% versus 7%, *P* < 0.05).

Figures 5(a) and 5(b) show reasons for missing exercise sessions ((a) 2 versus 12 min and (b) low versus high adherence). In both the 2 and 12 min groups, lack of time was a common reason for missing exercise sessions (29–37%). Among those with low adherence compared with high adherence significantly more participants ascribed lack of time (51% versus 17%, *P* < 0.001), difficulty in starting exercising after illness (26% versus 5%, *P* < 0.01), difficulty in starting exercising after a holiday (9% versus 0%, *P* < 0.05), and other various reasons (45% versus 19%, *P* < 0.001) to reasons for missing exercise sessions.

## 4. Discussion

The present summative process evaluation shows that while resistance training for neck-shoulder pain is generally well received among office workers, a one-size-fits-all approach may not be feasible for all employees. Using training diary registrations throughout the intervention and questionnaires at 10-week follow-up we were able to identify several important issues and characteristics of the intervention, which can be used to improve practical recommendations and design of future studies.

The training diary registrations (Figure 1) indicate high fidelity towards both interventions. The total number of repetitions per training session and progression of resistance reported in each group validate that the participants understood and complied with the intended interventions. Both groups approximately doubled their training resistance through the 10 weeks, which is similar to the progression reported in supervised strength training among office workers with trapezius myalgia [11]. Perceived exertion during training increased throughout the 10 weeks, which would be expected with increasing resistance and increased tolerance to pain. It is noteworthy that the 2 min group rated perceived physical exertion higher than the 12 min group. Thus, going to momentary muscular fatigue with as many repetitions as possible in the 2 min group was more exerting than performing more total repetitions with rest breaks in between in the 12 min group. This is important information for practical purposes as not all employees may enjoy the sensation of muscular exertion, which was also indicated by the replies in Figure 2 where many participants felt that the progression had been too fast.

As shown in Figure 1, approximately 4 of 5 participants completed more than 30 training sessions in 10 weeks (~3 times per week). The average training frequency of three times per week in the present study is in accordance with the recommendations from the American College of Sports Medicine; that is, untrained adults are recommended to perform resistance training three times a week [29]. However, approximately 1 of 5 participants performed less than 30 training sessions in 10 weeks, meaning that barriers for regular training also exist for some employees even with brief training programs.  Figure 5 shows that lack of time was the most common reason for missing exercise sessions, which is in agreement with several other studies [22, 31]. However, it may seem puzzling that lack of time can be a barrier for performing as little as 2 minutes of exercise. Other underlying factors in the work environment may explain this, for example, a stressful psychosocial work environment [32]. The context of interventions also matters, for example, support from management, resources, facilities, distance, and organizational culture [17, 31, 33–36]. In the present study, the upper management approved and supported that employees could participate during paid working hours. Further, the simplicity of the program made it possible for employees to train at the offices or in the hallways, which eliminated the extra time needed when going to a gym (transportation, changing clothes, etc.). Together these contextual factors may have facilitated the high adherence.

While many participants were satisfied with the program, the process evaluation revealed important points for practical recommendations. Figures 2 and 3 show participants feedback on the characteristics of the training program and type of training. Although most participants felt that the duration of the training sessions was appropriate, almost a third of the participants in the 12 min group felt that the training sessions were too long. By contrast, 14% in the 2 min group felt that the training sessions were too short. Considering that the 2 min and 12 min groups showed similar reductions of neck-shoulder pain and tenderness [13, 27], employees can freely choose the duration between 2 and 12 min per training sessions that they prefer or best fit their work schedule. Almost two-thirds of the participants would have liked to have more than one exercise to vary between, which may be important for long-term motivation. This suggests that the strategy of making the exercise program as simple as possible by including only a single exercise may not be optimal. Half of the participants with low adherence felt that the training frequency of five times per week was too much. While most participants felt that elastic resistance training was appropriate for them, 29% of those with low adherence would rather have trained something completely different. Altogether, these findings show that a one-size-fits-all approach may not be optimal for all employees. Importantly, general physical exercise including different types of activities has also shown to reduce neck-shoulder pain [10] but may be more time consuming. Thus, customization of physical exercise and individual preferences may be advisable when time and context allow this to increase motivation and long-term adherence.

Figure 4 shows participants feedback on supportive elements related to the training program. Most participants found the training diary and manual helpful and most of them found that a single instructional session was adequate. However, 9–18% of the participants felt that a single instructional session was too little, and 21% of the 12 min group had used email support. Based on the feedback from the therapist who managed the email support, the questions related mostly to confusions about sets and repetitions in the 12 min group. Thus, these factors should be carefully explained in future workplace interventions with resistance training to make sure that all participating employees understand basic resistance training concepts. It seems plausible that doing as many repetitions as possible in the 2 min group was easier to comprehend. Those with low adherence did not use the support more than those with high adherence, and it thus seems likely that low adherence was not due to a lack of understanding the program but rather due to the barriers shown in Figure 5, for example, lack of time or other underlying factors. Besides lack of time, Figure 5 also shows other important reasons for missing exercise sessions. Among those with low adherence, difficulties to start exercising after a holiday or illness were also reasons for missing exercise sessions. Thus, future workplace interventions may introduce concepts like “booster-sessions” once in a while to engage employees who have stopped training.

In conclusion, simple resistance training for neck-shoulder pain is generally well received among office workers, but a one-size-fits-all approach is not feasible for all employees. Based on the present study we recommend (1) including more than one exercise to vary between, (2) being aware that even 12-minute training and/or 5 times a week may be too much for some employees, and (3), when time and context allow, taking into account individual preferences to increase motivation and long-term adherence. Importantly, the contextual factors were good; for example, there were support and approval from the upper management to participate during paid working hours.

## Figures and Tables

**Table 1:** Demographics, clinical and work-related characteristics of the participants in the 2 and 12 min groups who replied to the follow-up questionnaire used in the present analyses. Values are reported as mean (SD) or percentage of participants.

	2-minute group	12-minute group
Number of participants	59	57
Demographics		
Age, year	44 (11)	43 (11)
Body mass index, kg*·*m^−2^	25 (5)	23 (4)
Percentage of women	88%	88%
Clinical		
Days with pain during previous year	180 (114)	194 (119)
Pain intensity in last 3 months (scale 0–10)	5.2 (1.9)	5.2 (2.1)
Work-related		
Computer use, percentage of work time	93 (14)	96 (11)
Weekly working hours	39 (5)	38 (5)
Duration of office work, years	11 (9)	10 (10)
